# The effect of music performance on the transcriptome of professional musicians

**DOI:** 10.1038/srep09506

**Published:** 2015-03-25

**Authors:** Chakravarthi Kanduri, Tuire Kuusi, Minna Ahvenainen, Anju K. Philips, Harri Lähdesmäki, Irma Järvelä

**Affiliations:** 1 Department of Medical Genetics, Haartman Institute, University of Helsinki, P.O. Box 720, 00014 University of Helsinki, Finland; 2 DocMus doctoral school, Sibelius Academy, University of the Arts, P.O. Box 30, FI 0097 Uniarts, Finland; 3 Department of Information and Computer Science, Aalto University, FI-00076 AALTO, Finland

## Abstract

Music performance by professional musicians involves a wide-spectrum of cognitive and multi-sensory motor skills, whose biological basis is unknown. Several neuroscientific studies have demonstrated that the brains of professional musicians and non-musicians differ structurally and functionally and that musical training enhances cognition. However, the molecules and molecular mechanisms involved in music performance remain largely unexplored. Here, we investigated the effect of music performance on the genome-wide peripheral blood transcriptome of professional musicians by analyzing the transcriptional responses after a 2-hr concert performance and after a ‘music-free' control session. The up-regulated genes were found to affect dopaminergic neurotransmission, motor behavior, neuronal plasticity, and neurocognitive functions including learning and memory. Particularly, candidate genes such as *SNCA*, *FOS* and *DUSP1* that are involved in song perception and production in songbirds, were identified, suggesting an evolutionary conservation in biological processes related to sound perception/production. Additionally, modulation of genes related to calcium ion homeostasis, iron ion homeostasis, glutathione metabolism, and several neuropsychiatric and neurodegenerative diseases implied that music performance may affect the biological pathways that are otherwise essential for the proper maintenance of neuronal function and survival. For the first time, this study provides evidence for the candidate genes and molecular mechanisms underlying music performance.

Music performance (typically playing an instrument) is a complex cognitive function of the human brain, whose biological basis is largely unknown. Performing music requires auditory and visual perception skills, attention, precise timing, extended control over movement, learning, memory and emotion^1^,^2^,^3^,^4^,^5^. Execution of such skills is essentially dependent on the bi-directional communication between the auditory and motor systems of the human brain^1^,^2^,^3^,^4^. Neuroscientific studies have demonstrated that musicians' brains exhibits structural and functional specializations compared to non-musicians^6^,^7^,^8^ and that music training induces neuroplasticity, including changes in the grey and white matter architectures, and cerebellar volume^5^,^9^,^10^. Moreover, by altering the brain's neural circuits and structural symmetry, music training has been reported to enhance cognitive performance, various forms of visual attention abilities, and mental abilities such as verbal and long-term memory, and reasoning^8^,^11^,^12^,^13^. Consistent training in music has also been shown to induce a commonality in the cognitive characteristics of professional musicians. For instance, in instrumentalists, practicing music leads to a shift from an effortful controlled cognitive processing to an effortless automatic cognitive processing^14^, thus leaving the limited attentional abilities available for higher-order processes of music performance^15^. Music performance is also known to induce emotion-related psychophysiological responses and generate a robust brainstem encoding of linguistic pitch patterns^16^,^17^. However, the molecular mechanisms and biological pathways mediating the effects of music performance so far remain unknown.

Genomic approaches enable the study of biological phenomena in an unbiased and hypothesis-free fashion, without prior knowledge about the biological background of the phenotype of interest^18^. Here, we have investigated the effect of music performance on human peripheral blood transcriptome of professional musicians during a 2-hour concert session and in a 2-hour session without music exposure.

## Results

### Statistical comparisons

The general characteristics of the participants are shown in Table 1. We assessed the differences between the two groups using statistical analyses performed in R, a statistical computing platform. We used a two-sided t-test for continuous variables and a two-sided Fisher's exact test for count variables. At a conventional significance level of 5%, the two groups neither differed in the general characteristics (age and gender) nor in the task-related characteristics (current practicing hours, age at the commencement of training, music education and instrument). This suggests that the two groups are sufficiently similar for the comparison of transcriptional responses.

### Transcriptional response after music performance

**Figure 1:**
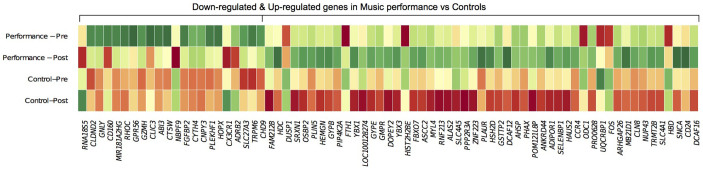
Differential gene expression of music performance vs ‘music-free' controls. Heat plot representation of mean expression values of music performance (pre, post) vs control session (pre, post). Red-yellow-green palette represents low-moderate-high expression values.

To identify the differentially expressed genes, we compared the magnitude of pre-post fold-changes in the genome-wide transcriptional profiles of the participants in the concert performance (n = 10) and in the control session (n = 10). RankProd non-parametric statistics and a pre-specified effect-size cut-off (>1.2 fold-change over time across conditions, and at least a pre-post change of 15% in gene expression in the concert performance session, pfp 0.05) identified 73 differentially expressed genes (51 genes relatively up-regulated and 22 genes relatively down-regulated). The differentially expressed genes and the individual fold-changes of all the genes are listed in Table S1 and a heat plot comparison of the pre-post changes in both conditions is shown in Figure 1.

Gene ontology classification (Table S2) revealed that the genes up-regulated after music performance are involved in the uptake, transport, and regulation of neurotransmitters (*CLN8, SNCA*), catecholamine biosynthetic process, (*HDC, SNCA*), elevation of cytosolic calcium ion concentration (*CCR4, CD24, SNCA*), cellular iron ion homeostasis (*FTH1, ALAS2*), the hemoglobin metabolic process (*AHSP, ALAS2*), associative learning (*CLN8, FOS*), and motor behavior (*CCR4, CLN8, PLAUR, FOS, SNCA*). Additionally, music performance also resulted in the up-regulation of the response genes of cAMP (*DUSP1, FOS*), oxidative stress (*SRXN1, DUSP1, CLN8, FOS, SNCA*), chemical stimulus (*SRXN1, CCR4, DUSP1, CLN8, PLAUR, ALAS2, ADIPOR1, CD24, FOS, SNCA*), and biotic stimulus (*CCR4, HIST2H2BE, ODC1, CD24, FOS, SNCA*).

Furthermore, Entrez gene annotation and an extensive literature survey revealed that the genes up-regulated after music performance include several genes that are involved in dopamine neuronal homeostasis (*SNCA, FBXO7, PIP4K2A, PPP2R3A*), synaptic plasticity (*SNCA, FOS, CLN8, PIP4K2A*), learning, memory and cognitive functions (*FOS, HDC, CLN8, FTH1, DOPEY2*), neurotransmission (*DUSP1, FBXO7, PPP2R3A*), neurite outgrowth and neurogenesis (*CD24, SELENBP1*), neuronal differentiation (*PLAUR, CLN8*), neuronal activity (*SLC4A1, SLC4A5, HIST2H2BE*), calcium ion homeostasis (*FOS, CLN8, MYL4*), glutathione metabolism (*ODC1, PIP4K2A*), speech and language (*DOPEY2, RNF213, ANKRD44*), and neuropsychiatric and neurodegenerative diseases (*SNCA, FOS, ARHGAP26, HDC, CLN8, SELENBP1, FTH1, ADIPOR1, FBXO7, PIP4K2A, SRXN1, DOPEY2, GMPR, RNF213*). Interestingly, some of the up-regulated genes include biomarkers of song perception and production in songbirds (*SNCA, FOS, DUSP1, ZNF223, ARHGAP26, PLAUR, SELENBP1, FTH1, SRXN1, ASCC2*) (Table 2, Table S3). Down-regulated genes are known to be involved in cellular defense response (*CD160, CX3CR1, GNLY*). Based on Entrez gene annotation, genes involved in G-protein coupled receptor protein signaling (*GPR56, ADRB2, CX3CR1*) were also found to be down-regulated.

### Upstream regulators

Upstream transcription regulator analysis was performed to identify the molecules that might mediate the observed differences in gene expression. These results show that the up-regulated genes significantly overlap the known target genes of transcription regulators such as *GATA1* (p-value 0.000003; Z-score 2.000), cytokines *CCL5* and *TNFSF11* that are involved in glucocorticoid regulation (p-values 0.00002, 0.00023; Z-scores 1.969, 2.150), and insulin like growth factor *IGF1* (p-value 0.0017; Z-score 2.149). On the other hand, down-regulated genes significantly overlapped the known target genes of transcription regulators that include pro-inflammatory cytokines *IL15* and *IL2* (p-values 0.000013, 0.000062; Z-scores −0.740, −1.547) (Table S4).

*GATA* transcription factors *GATA-1* and *GATA-2* have been demonstrated to regulate the expression of *SNCA* and its co-expression network of 35 genes^19^. We checked if music performance affected the GATA-regulated *SNCA* co-expression network. Interestingly, 9/35 (25.7%) of the *SNCA* co-expressed genes were found to be differentially expressed in our study. This suggests that *GATA* transcription factors might be the candidate transcription regulators of the observed differential expression.

### Functional interactions

**Figure 2:**
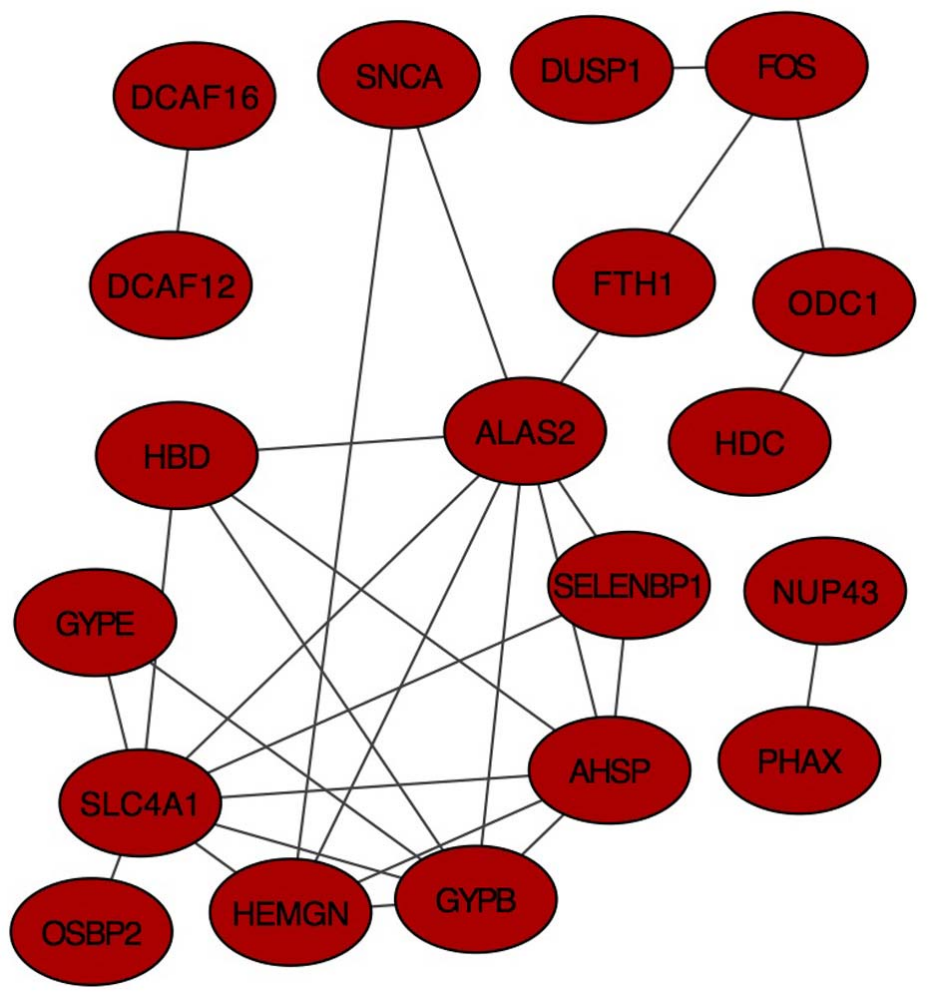
Network of known functional interactions among the up-regulated genes. We used the STRING database^71^ to explore the known functional interactions among the up-regulated genes. STRING database integrates known and predicted protein interactions that are compiled from multiple sources based on high-throughput experiments, computational prediction methods, co-expression, and previous knowledge. The genes belonging to this functional network may represent the likely candidate genes that mediate the effects of music performance. Nodes represent genes and the edges represent the known functional interactions between the genes.

Further, we used the STRING database to mine out the known functional interactions (Figure 2). Among the up-regulated genes, we found a significant functional network with 29 known interactions and 6.37-fold increased interactions than expected (p-value: 1.77 × 10^−14^).

## Discussion

This study demonstrates that music performance affects the gene expression profiles in professional musicians. A plethora of functional neuroimaging studies have demonstrated that playing and listening to music have multiple measurable effects on human brain structure and function^3^,^20^,^21^,^22^,^23^,^24^, and the wide range of biological mechanisms found in this study may explain the likely molecular evidence underlying some of those effects.

The up-regulation of dopaminergic neurotransmission-related genes is consistent with the findings of functional neuroimaging studies that have earlier demonstrated the endogenous release of dopamine during music listening^21^,^25^. Of particular importance, the up-regulated gene *alpha-synuclein* (*SNCA*), which maintains dopamine neuronal homeostasis^26^, has earlier been identified as a strong candidate for musical aptitude on chromosome 4q22.1^27^ and has been demonstrated to be regulated in the song control system of songbirds^26^,^28^. The co-expression network of *SNCA* (35 genes) that affects heme metabolism^19^ is known to underlie the dysfunction of iron ion homeostasis observed in Parkinson's disease. Interestingly 25.7% (9/35) of the *SNCA*'s co-expression network, which affects heme metabolism and iron ion homeostasis^19^, was found to be up-regulated after music performance along with *SNCA* suggesting that music performance may modulate the biological pathways that are otherwise essential for the proper maintenance of structure, function and survival of neurons^29^.

The up-regulation of several motor behavior-related genes may elucidate the molecular pathways that mediate the execution of fine motor skills such as timing, sequencing, and spatial organization of movement, which are essential for playing and performing music^1^,^8^. As motor behavior is primarily controlled by dopaminergic neurotransmission^30^,^31^, the genes related to both dopaminergic neurotransmission and motor behavior may act in harmony during music performance.

The up-regulation of genes related to neurite outgrowth, neurogenesis and neurotransmission is in agreement with the plethora of the neuroscientific literature, which demonstrated that practicing music induces neuronal plasticity^2^,^3^,^5^,^24^ and neurogenesis^32^. The up-regulated genes that affect synaptic function may explain the enhanced synaptic plasticity observed in professional musicians^33^. Some of the up-regulated genes related to learning, memory and cognitive functions may be induced by training in music^2^,^3^,^8^,^34^,^35^. However, while interpreting the results, we cannot exclude the effect of genetic component on gene expression in professional musicians. For instance, our previous genome-wide linkage and association study of musical aptitude has identified several genetic loci that are associated with musical aptitude, suggesting a genetic effect. The loci contain genes responsible for inner ear development, auditory pathways and neurocognitive processes that underlie musical aptitude^27^. We propose that the ability to enjoy and practice music requires musical aptitude, which is a common and innate trait. The drive for music is facilitated by musical aptitude and seldom arises without exposure to music in musically rich environments. Secondly, the results may be due to the general cognitive abilities that have been shown to be genetically determined^36^,^37^.

We also identified genes that are involved in the elevation of cytosolic calcium ion concentration and calcium ion homeostasis. It is known that stimulation of the auditory system elevates the outer hair cell calcium ion concentration^38^ and calcium ion concentration essentially regulates neurotransmitter release^39^, synaptic transmission^40^, activity-dependent synaptic plasticity^41^ and gene expression^42^. For example, intracellular calcium is thought to regulate neuronal firing pattern, which controls song behavior in songbirds^43^. These data allows us to speculate that calcium ion homeostasis may play a vital upstream role in music-induced dopamine release^21^,^25^, synaptic plasticity^33^ and transcriptional alterations.

In addition, disrupted/mutated forms of several of these genes (*SNCA, FOS, ARHGAP26, HDC, CLN8, SELENBP1, FTH1, ADIPOR1, FBXO7, PIP4K2A, SRXN1, DOPEY2, GMPR, RNF213, DCAF16, DCAF12*) have been implicated in various neuropsychiatric and neurodegenerative diseases (Table 2; detailed in Table S3). We hypothesize that the modulation of the genes related to neuropsychiatric and neurodegenerative diseases by music performance may at least partially explain the effect of music as a therapeutic tool in clinical settings^44^.

**Figure 3:**
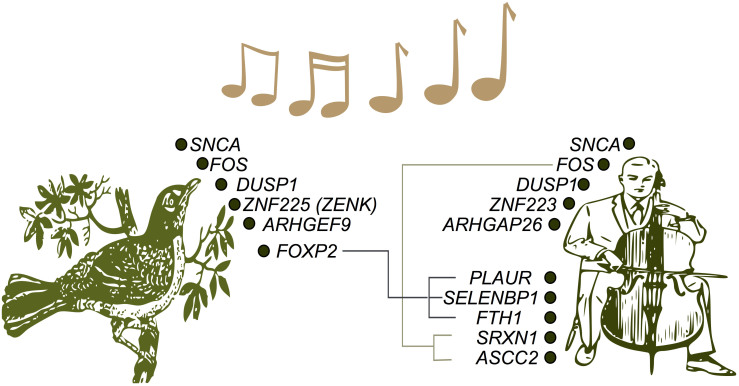
Evolutionary conservation of music perception/production. The genes up-regulated after music performance such as *SNCA, FOS*, and *DUSP1* have been demonstrated to be regulated in the song control system of songbirds^26^,^28^,^47^,^48^,^49^ whereas *ZNF223* and *ARHGAP26* have been known to be functionally similar to *ZNF225 (ZENK)* and *ARHGEF9* that are regulated during song perception and production in songbirds^50^,^72^,^73^. The up-regulated genes *SRXN1* and *ASCC2* are the known target genes of *FOS*. The up-regulated genes *PLAUR, SELENBP1* and *FTH1* are the known direct target genes of *FOXP2*. *FOXP2* gene has been known to be a very important candidate gene for song and speech development. Reduced activity of *FOXP2* has been known to interfere with dopaminergic modulation of vocal variability, thus impairing song and speech development^74^. The vector graphics of songbird and cello player have been obtained from Openclipart (https://openclipart.org/) and modified.

It is noteworthy that modern humans share an identically functioning auditory center with the first primates that lived millions of years ago^45^ suggesting high evolutionary conservation of sound perception. More recently, a wide- spread adaptive convergent sequence evolution has recently been found in echolocating bats and dolphins^46^, implicating numerous genes linked to hearing and vision, of which, *protocadherin 15* (*PCDH15*) was found to be associated with musical aptitude in our GWA study^27^. In this study, we found the up-regulation of several genes such as *SNCA, FOS* and *DUSP1* that have been demonstrated to be regulated during song perception and production in songbirds (Figure 3). Both *FOS* and *DUSP1* have been described as the immediate early response genes (IEGs) that govern the motor-driven gene expression in songbirds during singing^47^,^48^,^49^,^50^. Various types of stimuli including neuronal excitation^51^ and auditory stimulation^52^ induce *FOS*, where it acts as a bridge between synaptic transmission and gene expression^51^,^53^. Interestingly, we found the up-regulation of several direct targets of *FOS* such as *SRXN1, ASCC2, FTH1, ODC1, PLAUR*, and *SELENBP1*. A recent study by Pfenning et al.^54^ compared the brain transcriptomes of songbirds and humans and identified convergent gene expression specializations in multiple genes related to motor behavior, speech production, learning and memory. Approximately 30% (22/73) of the differentially expressed genes that were detected in our study (including *SNCA*) belong to the gene families that have been shown to contribute significantly to shared gene expression specializations in the brains of humans and song-learning birds (Table S5). Another recent study by Whitney et al.^55^ analyzed the genome-wide singing-regulated gene expression across time in four major brain regions of songbirds and reported a total of 1883 singing-regulated genes, with *FOS* being the most significant gene. Several genes that were differentially expressed here after music performance (*FOS, PLIN5, ODC1, DUSP1, FBXO7, HIST2H2BE, DOPEY2, and PHAX*) have also been reported by Whitney et al. to be regulated by singing in songbirds. Furthermore, studies in songbirds have also revealed the role of *FOXP2* in song learning and singing^56^,^57^. Here, we did not find any differential activity of *FOXP2*, but its target genes were found to be up-regulated (*PLAUR, SELENBP1, FTH1*).

The upstream regulator analysis and the co-regulation of several genes belonging to the *GATA*-regulated gene network suggested that *GATA* transcription factors could be the candidate upstream regulators of the observed transcriptional alterations (e.g. *ADIPOR1, AHSP, ALAS2, FBXO7, GMPR, GYPB, GYPE, HBD, PIP4K2A, SELENBP1, SNCA, SLC4A1*). Interestingly, *GATA2*, which is located in the most significant region of association with musical aptitude (at 3q21)^27^, is abundantly expressed in dopaminergic neurons and binds to the intron-1 of endogenous neuronal *SNCA* to regulate its expression.

Peripheral whole blood shares more than 80% of its transcriptome with several other tissues including the brain^58^,^59^, which enabled the use of peripheral blood as a window for transcriptomic alterations in the brain with great success^60^,^61^. Although, recruitment of professional musicians into the study and arranging the experiment sessions is easier said than done, we managed to recruit a sufficiently decent sample set that is comparable to similar studies^62^,^63^.

Here we investigated the transcriptional alterations in professional musicians after music performance. Rather, to be able to comprehensively demonstrate the professional musician-specific transcriptional alterations after music performance, new studies are required to study professional musicians, non-professional musicians and non-musicians using multiple study settings. Studies are required to assess the effect of playing different genres of music, at different ages, using different surroundings (with and without audience), and with varying durations of the performance. The definition of phenotypes would be crucial in such studies. For instance, some non-professional musicians may have substantial education/training in music, yet their profession could be different. We hypothesize that there will be differences and similarities in the transcriptional responses of non-professional musicians and non-musicians after music performance. Differences are likely to be caused because of differences in genetic background and environmental exposure to music among the study groups. Similarities are likely to be seen because of the common evolutionary background of sound perception in mammalians^45^,^46^,^54^,^55^.

The findings may provide a valuable background for molecular genetic studies of music evolution, the development of language, the neurobiological background of emotions, neurological and neuropsychiatric diseases and attempts to understand the molecular mechanisms that mediate the effects of music therapy.

## Methods

### Ethics statement

The Ethical Committee of Helsinki University Central Hospital approved the study. Written informed consent was obtained from all the participants. The methods were carried out in accordance with the approved guidelines.

### Concert performance

A total of 13 musicians participated in the performance part of the study. The participants belonged to Tapiola Sinfonietta, a chamber orchestra of 42 instrumentalists. Of them, samples from 10 participants (3 male, 7 female, median age: 49) were found eligible for the study. Two participants were excluded owing to the data quality, while one participant was excluded because of her relatively shorter duration of performance. The majority of the participants (9) played string instruments (violin, 6; viola, 2; cello, 1), whereas one played the flute. The study was performed during one of the concerts belonging to their program. The musicians played the following pieces: I. Stravinsky: Apollon musagète (for string orchestra), J. Haydn: “Deh soccorri un'infelice” from the opera La fedeltà premiata, L. Cherubini: “Ah! nos peines seront communes” from the opera Médée, J.C. Bach: “Ch'io parta” from the opera Temistocle and W.A. Mozart: Symphony nr 40, g-minor. Peripheral blood samples were collected from all the participants just before and immediately after the concert that lasted about two hours.

Data about the participants' activities before the concert (e.g., previous night's sleep, caffeine, alcohol, working during the day), stress factors (e.g., travel to work, nervousness), and personal opinions (familiarity of the music played, the impact of the conductor, pleasantness of the event) were collected using a questionnaire. The responses did not show any significant differences between the participants (data not shown).

### Control study

Ten professional musicians (2 male, 8 female, median age 40.5) participated in the control study. All the samples were found eligible for the analyses. Four of the participants were violinists and three pianists; one played the horn, one the bassoon and one the flute. The control session was performed at the Sibelius Academy, University of Arts, Helsinki, in a “music-free” environment and lasted 2 hours (same duration as the music performance). During the control study the participants were taking a walk outside or listening to a lecture. Peripheral blood samples were collected from the participants just before and immediately after 2 hours in the control session.

### Genome-wide expression profiling

We used PAXgene blood RNA tubes (PreAnalytiX GmbH, Hombrechtikon, Switzerland) as per the kit instructions for the collection of peripheral blood samples (2 × 2.5 ml) in both the sessions. Further, we used PAXgene blood miRNA Kit (PreAnalytiX GmbH, Hombrechtikon, Switzerland) as per the kit instructions for the isolation of total RNA. Next, we tested the purified RNA samples for purity and concentration using the NanoDrop 1000 v.3.7 (Thermo Fisher Scientific, USA). In addition, we used the Ambion's Human GLOBINclear^TM^ kit (Applied Biosystems, USA) as per the kit insert, for the depletion of globin mRNA. Further, we used the 2100 Bioanalyzer (Agilent Technologies, Germany) to measure the RNA integrity of the samples, before diluting to 50 ng/μl using RNase-free water. A total of 2 μg of RNA was assayed on the Illumina HumanHT-12 v4 bead array (Illumina Inc.; San Diego, CA, USA), which targets more than 47,000 probes.

### Bioinformatics

We used Lumi bioconductor package to preprocess the data, which included background correction, variance stabilizing transformation, and quantile-normalization. Next, we used the geneFilter bioconductor package to filter out the duplicate and un-annotated probes. Further, probes that have a lower intensity when compared to the background signal were filtered out using the Illumina's detection p-value threshold of 0.01. Next, we retained only those probes that were expressed in at least half of all the arrays (concert and control sessions) for the further analyses. We identified the differentially expressed genes by comparing the magnitude of pre-post changes in gene expression across conditions using the rank product non-parametric method implemented in the RankProd bioconductor package^64^. Rank product provides a useful non-parametric method to identify differentially expressed genes with reliable significance thresholds, when heterogeneity exists within and between groups^65^. This statistically rigorous and biologically motivated test detects the genes that are consistently ranked high among the most up- or down-regulated genes across all the samples, irrespective of the heterogenity in replicate experiments^65^. Because of this reason, rank product method has been successfully used to perform meta-analyses to combine datasets generated from different origins, laboratories and environments^64^. Moreover, rank product method has been known to outperform all the other popular methods like empirical bayes statistic (limma) and SAM when the sample size is small and when there are high levels of noise in the dataset^66^,^67^. However, this conservative approach identifies only the most consistent biological signal. Unlike the comparison of absolute gene expression values, comparison of fold-changes over time, across conditions, reduces the effect of other confounding factors. After the identification of differentially expressed genes using a pfp (estimated percentage of false positive predictions) of 0.05 in RankProd, we selected only those genes that exceeded an effect-size cut-off (>1.2 fold-change over time across conditions, and at least a pre-post change of 15% in gene expression in the concert performance session). Here, two aspects of selecting the differentially expressed genes are noteworthy. First, the estimated percentage of false positive predictions employed by RankProd is also known as false discovery rate, and is equivalent to the conventional FDR method^64^. Second, there exists a widespread misconception that only two-fold changes are significant^68^ and that false notion is based on the very initial publications of microarray studies, which used a two-fold change criteria for a particular group of experiments owing to biological relevance. Fold-change thresholds are completely arbitrary and in the majority of the cases they depend upon the underlying biological question. For example, studies that investigated the effect of gene-environment interactions (socio-environmental effect^62^, yogic meditation effect^69^) used unorthodox fold-change thresholds. Further, we chose to perform gene ontology classification using the over-representation analysis implemented in GeneTrail^70^ because of the homogeneous fold-change distribution of all the differentially expressed genes. This method uses a hypergeometric distribution test along with a conservative multiple testing correction method (FDR < 0.05), to assess whether genes belonging to certain functional categories are overrepresented in the dataset. In addition, we performed upstream transcription regulator analysis using IPA (Ingenuity® Systems), which essentially predicts all the upstream transcription regulators (transcription factors, receptors, cytokines, microRNA, kinases) that could have possibly mediated the observed differential expression. Based on the overlap between known targets of a transcription regulator and the set of differentially expressed genes, an overlap p-value is computed using Fisher's exact test (p < 0.01). Further, the activation states of the predicted transcription regulators are also inferred using an activation Z-score, which is based on literature-derived knowledge on the direction of regulation (either activating or inhibiting). Further, we also performed a functional interaction analysis using STRING database^71^ to understand and assess the degree of protein-protein interactions among the set of differentially expressed genes. STRING database is a unique resource that provides a global perspective of protein-protein interactions. It contains data that is curated from high throughput experiments, computational predictions and transferred interactions, and also interactions obtained through text mining.

## Supplementary Material

Supplementary Information Table S1

Supplementary Information Table S2

Supplementary Information Table S3

Supplementary Information Table S4

Supplementary Information Table S5

## Figures and Tables

**Table 1:** General characteristics of the participants

Characteristic	Concert performance (N = 10)		Control (N = 10)		p
	N	Median	N	Median	
Age		49		40.5	0.054
Age at the commencement of training		6		6	0.230
Female	7		8		0.999
String instrumentalists	9		4		0.057
Wind instrumentalists	1		3		0.582
Keyboard instrumentalists	0		3		0.210
University degree ≧ master's/diploma	9		8		1.000
Training hours per day (currently)		5		5	0.221

**Table 2:** Putative biological functions of the differentially expressed genes

Putative Biological Function	Genes
Implicated in song perception and production in songbirds	*SNCA, FOS, DUSP1*
Functionally similar to genes implicated in song perception and production in songbirds	*ZNF223, ARHGAP26*
Direct targets of FOXP2	*PLAUR, SELENBP1, FTH1*
Direct targets of FOS	*SRXN1, ASCC2, FTH1, ODC1, PLAUR, SELENBP1*
Dopamine neuronal homeostasis	*SNCA, FOS, FBXO7, PIP4K2A, PPP2R3A*
Synaptic function	*SNCA, FOS, CLN8, PIP4K2A*
Learning, memory, and cognitive functions	*FOS, HDC, CLN8, FTH1, DOPEY2*
Neurotransmission	*DUSP1, FBXO7, PPP2R3A*
Neuroprotection	*SNCA, FOS, ADIPOR1, SRXN1*
Calcium ion homeostasis	*FOS, CLN8, MYL4*
Neurite outgrowth and neurogenesis	*CD24, SELENBP1*
Neuronal differentiation	*PLAUR, CLN8*
Neuronal activity	*SLC4A1, SLC4A5, HIST2H2BE*
Glutathione metabolism	*ODC1, PIP4K2A*
Speech and language	*DOPEY2, RNF213, ANKRD44*
Neuropsychiatric and neurodegenerative diseases	*SNCA, FOS, ARHGAP26, HDC, CLN8, SELENBP1, FTH1, ADIPOR1, FBXO7, PIP4K2A, SRXN1, DOPEY2, GMPR, RNF213, DCAF16, DCAF12*
