# Supplementary material for: The effect of music performance on the transcriptome of professional musicians
Source: Sci Rep. 2015 Mar 25;5:9506. doi: 10.1038/srep09506 (PMC5380155; doi:10.1038/srep09506)
Supplement: Supplementary Information — Table S3 [file srep09506-s3.pdf]

The effect of music performance on the transcriptome of professional musicians

Chakravarthi Kanduri[1], Tuire Kuusi[2], Minna Ahvenainen[1], Anju K Philips[1], Harri Lähdesmäki[3], Irma Järvelä[1]

(1) Department of Medical Genetics, Haartman Institute, University of Helsinki, P.O. Box 720, 00014 University of Helsinki, Finland

(2) DocMus doctoral school, Sibelius Academy, University of the Arts, P.O. Box 30, FI 0097 Uniarts, Finland

(3) Department of Information and Computer Science, Aalto University, FI-00076 AALTO, Finland

Table S3: Candidate genes involved in music performance and their known biological functions

Gene	Known Biological Functions
SNCA	Involved in dopamine (DA) neuronal homeostasis (1, 2); Regulates the maintenance of mature synapses, and the stabilization of synaptic function (2). Essential for synaptic plasticity via the genesis and maintenance of presynaptic vesicles (3). The normal function of *SNCA* is regulated by neural activity and the response of *SNCA* depends upon the stimulus intensity (4). Described as the protein that is regulated during a critical period of song learning in songbirds (5); Located on chromosome 4q22.1 that shows significant linkage to musical aptitude (6, 7); Furthermore, *GATA2*, which is located in the most significant region of association with musical aptitude at 3q21 (6), is abundantly expressed in dopaminergic neurons and binds to intron-1 of endogenous neuronal *SNCA* to regulate its expression (8); Reduces oxidative stress and has a neuroprotective role by weakening the dopamine transporter activity (9)
FOS	A phosphoprotein and transcription regulator; One of the immediate-early response genes in the brain; Transiently induced by various types of stimuli including neuronal excitation (10, 11); Acts as a bridge between synaptic transmission and alterations in gene expression (12). Particularly, the intermediary role of FOS in coupling dopaminergic synaptic transmission and neuropeptide

	gene expression has been evident; May have a role in learning and memory processes (10, 11); Stimulation/activation of auditory cortex and exposure to glutamate elevated the FOS expression in auditory thalamus (13–15). Acts as neuroprotector (16); The activity of FOS gene is controlled by calcium and disruption of calcium homeostasis suppresses FOS activity, which induces neurodegeneration (17).
DUSP1	Another immediate-early response gene; Known to be an important regulator in human cellular response to environmental stress and affects MAPK signaling pathway by dephosphosphorylating MAPK (18, 19); Induced by growth factors, glucocorticoids, oxidative stress and heat shock (20); Induction of DUSP1 gene by glucocorticoids inhibits inflammation (21, 22); Localized in discrete neuronal populations within the brain suggesting that it could play a key role in neurotransmission (23).
PLAUR	Importantly, regulated by FOXP2, a gene that has been implicated in song learning and singing in songbirds (24, 25). PLAUR activation modulates variety of intracellular signaling pathways such as diacylglycerol accumulation, modulation of cAMP levels, calcium modulation, inositol phosphate turnover, activation of tyrosine kinase and serine/threonine kinases (26–32). Expression associated with neuronal differentiation;
ARHGAP26	Belongs to a family of Rho GTPase- activating proteins that regulate RHOA and CDC42. A gene from the same gene family, with similar function, has been found to be regulated during singing in songbird (33). The expression of this was also found to be

	decreased in the X-linked α thalassemia mental retardation syndrome (34).
HIST2H2BE	Changes in the activities of histone family of proteins have been shown to shape the transcriptional responses to neuronal activity. Histone modifications have been implicated in stimulus-dependent regulation of transcription. (35). Specifically, HIST2H2BE has been shown to display activity-dependent expression (36).
ZNF223	A C_2H_2 type zinc finger protein with no known function. Interestingly, another C_2H_2 type zinc finger protein, ZNF225 (ZENK; EGR1) has been repeatedly described as the immediate-early response gene in songbirds during song learning and singing. Along with FOS, ZENK is the most well-documented gene in songbirds (33, 37–40).
HDC	Rate-limiting enzyme in histamine biosynthesis; Histamine system in the brain is mainly involved in alerteness, arousal, cognition, learning and memory; A disruption in the brain's histaminergic system has been implicated in several neurological and neuropsychiatric diseases including Alzheimer's, Parkinson's, Schizophrenia and Tourette's syndrome (41–43).
CLN8	Important candidate gene for associative learning; CLN8 deficiency leads to poor associative learning (44), multiple behavioral abnormalities, especially locomotory behavior, poor memory and heightened aggression (45). In synaptic vesicle pathway, CLN8 regulates the glutamate receptor activity and also the synaptosomal glutamate reuptake (46). Decline in synaptosomal glutamate reuptake leads to glutamatergic excitotoxicity, which disrupts calcium ion homeostasis (47, 48). CLN8 also plays a role in cell

	proliferation during neuronal differentitation and also protects against cell death (49).
CD24	A glycosylphos-phatidylinositol-anchored membrane glycoprotein; Known to inhibit neurite outgrowth and affects adult neurogenesis; Participates in signaling events occuring during neuronal migration (50–52).
SLC4A1	Belongs to electrogenic sodium bicarbonate cotransporter family (NBC); Activated during neuronal activity; Genes of NBC family are known to regulate intracellular and extracellular pH during neuronal excitation and activation. Neuronal activity causes an increase in the extracellular potassium concentration leading to glial depolarization, which then activates sodium bicarbonate cotransporters (53).
SLC4A5	Belongs to electrogenic sodium bicarbonate co-transporter family (NBC); Activated during neuronal activity; Genes of NBC family are known to regulate intracellular and extracellular pH during neuronal excitation and activation. Neuronal activity causes an increase in the extracellular potassium concentration leading to glial depolarization, which then activates sodium bicarbonate co-transporters (53).
ODC1	Rate-limiting enzyme that controls polyamine biosynthesis pathway and is involved in glutathione metabolism through the modulation of glutamate receptors (54, 55).
SELENBP1	Importantly, regulated by FOXP2, a gene that has been implicated in song learning and singing in songbirds (24, 25). Neurogenic

	factor that promotes neurite outgrowth (56); Elevated in schizophrenia to compensate and restore the neuronal connectivity and functioning (57).
FTH1	Importantly, regulated by FOXP2, a gene that has been implicated in song learning and singing in songbirds (24, 25). FTH1 gene is involved ion channel activity and plays a crucial role in iron homeostasis within the brain, and thus cognitive functions (58). Defects in ferritin proteins are associated with several neurodegenerative diseases (58). Up-regulation of iron-regulating genes has been correlated with cognitive functions (59).
ADIPOR1	Neuroprotective role in amyloid-beta neurotoxicity in Alzheimer's disease (60) and also known to improve cognitive decline in dementia (61).
FBXO7	A gene known to be involved in synucleinopathies along with SNCA gene; Highly expressed in the brain; crucial for dopaminergic neuronal function, neurotransmission, and dopamine-dependent locomotor activity; Loss of this gene leads to dopaminergic neuronal degeneration; Also shown to be a negative regulator of NFKB (62–64).
PIP4K2A	Highly expressed in the brain; Mutations in this gene are implicated in 10p-linked psychiatric disorders such as Bipolar and Schizophrenia (65); Involved in receptor-activated signal transduction, ion channel function and synaptic vesicle function (66–69); Especially involved in the synthesis of PIP_2, a precursor to second messengers in phosphoinositide signal transduction

	cascade, and a target for mood stabilizing drugs (70). Mutation in this gene has been associated with reduced glutamate uptake by excitatory amino acid EEAT3, and this altered EEAT3-induced glutamate metabolism has been implicated in Schizophrenia (71). Also a variant of this gene is known to suppress the activity of dopaminergic neurons (72).
PPP2R3A	This gene, highly expressed in the striatum, is known to integrate the effects of dopamine and other neurotransmitters (73). This protein phosphatase subunit dephosphorylates the major target of dopamine, DARPP-32, through cAMP- and Ca^{+2} dependent mechanisms to integrate neurotransmission (73).
SRXN1	Regulated by FOS gene; Neuroprotective and antioxidant gene that is regulated by FOS in response to synaptic activity (74, 75).
ASCC2	Enhances transactivation of FOS gene (76).
DOPEY2	Has been shown to be an important candidate gene for learning, memory, general intelligence and language abilities. Repeatedly implicated in Down's syndrome and intellectual disability characterized by learning defects and cognitive decline (77–80).
GMPR	Has been implicated in major depressive disorder and Autism spectrum disorder (81, 82)
RNF213	Repeatedly implicated in Moyamoya disease, a cerebrovascular disease that occurs in basal ganglia of brain and is characterised by disturbed consciousness, speech deficits (usually aphasia), sensory and cognitive impairments, involuntary movements, and vision problems (Entrez gene ID: 57674).

ANKRD44	Suggestive association with dyslexia, a language disorder that shares genetic background with music perception and song learning (83).
DCAF16	Substrate receptors for *CUL4-DDB1 E3* ubiquitin ligase complex and regulates histone methylation (84). Among the two *CUL4* paralogs, deficits in *CUL4B* has been linked to X-linked intellectual disability. In particular, *CUL4B* gene has been demonstrated to regulate the number of paravalbumin-positive *GABA*ergic neurons and dendritic properties in hippocampus (85).
DCAF12	Substrate receptors for *CUL4-DDB1 E3* ubiquitin ligase complex and regulates histone methylation (84). Among the two *CUL4* paralogs, deficits in *CUL4B* has been linked to X-linked intellectual disability. In particular, *CUL4B* gene has been demonstrated to regulate the number of paravalbumin-positive *GABA*ergic neurons and dendritic properties in hippocampus (85).
MYL4	Involved in calcium ion binding (Entrez gene ID: 4635).

References

1. Oczkowska A, Kozubski W, Lianeri M, Dorszewska J (2013) Mutations in PRKN and SNCA Genes Important for the Progress of Parkinson’s Disease. *Curr Genomics* 14:502–17. Available at: http://www.ncbi.nlm.nih.gov/pubmed/24532983 [Accessed April 24, 2014].

2. Murphy DD, Rueter SM, Trojanowski JQ, Lee VM (2000) Synucleins are developmentally expressed, and alpha-synuclein regulates the size of the presynaptic vesicular pool in primary hippocampal neurons. *J Neurosci* 20:3214–20. Available at: http://www.ncbi.nlm.nih.gov/pubmed/10777786 [Accessed April 8, 2014].

3. Abeliovich A et al. (2000) Mice lacking alpha-synuclein display functional deficits in the nigrostriatal dopamine system. *Neuron* 25:239–52. Available at: http://www.ncbi.nlm.nih.gov/pubmed/10707987 [Accessed April 8, 2014].

4. Fortin DL et al. (2005) Neural activity controls the synaptic accumulation of alpha-synuclein. *J Neurosci* 25:10913–21. Available at: http://www.ncbi.nlm.nih.gov/pubmed/16306404 [Accessed April 2, 2014].

5. George JM, Jin H, Woods WS, Clayton DF (1995) Characterization of a novel protein regulated during the critical period for song learning in the zebra finch. *Neuron* 15:361–72. Available at: http://www.ncbi.nlm.nih.gov/pubmed/7646890 [Accessed April 3, 2014].

6. Oikkonen J et al. (2014) A genome-wide linkage and association study of musical aptitude identifies loci containing genes related to inner ear development and neurocognitive functions. *Mol Psychiatry*. Available at: http://www.ncbi.nlm.nih.gov/pubmed/24614497 [Accessed March 19, 2014].

7. Pulli K et al. (2008) Genome-wide linkage scan for loci of musical aptitude in Finnish families: evidence for a major locus at 4q22. *J Med Genet* 45:451–456. Available at: http://www.ncbi.nlm.nih.gov/pubmed/18424507.

8. Scherzer CR et al. (2008) GATA transcription factors directly regulate the Parkinson's disease-linked gene alpha-synuclein. *Proc Natl Acad Sci U S A* 105:10907–12. Available at: http://www.pubmedcentral.nih.gov/articlerender.fcgi?artid=2504800&tool=pmcentrez&rendertype=abstract [Accessed April 24, 2014].

9. Wersinger C, Sidhu A (2003) Attenuation of dopamine transporter activity by alpha-synuclein. *Neurosci Lett* 340:189–92. Available at: http://www.ncbi.nlm.nih.gov/pubmed/12672538 [Accessed April 8, 2014].

10. Morgan JI, Curran T (1991) Stimulus-transcription coupling in the nervous system: involvement of the inducible proto-oncogenes fos and jun. *Annu Rev Neurosci* 14:421–51. Available at: http://www.ncbi.nlm.nih.gov/pubmed/1903243 [Accessed June 29, 2014].

11. Kaczmarek L, Nikołajew E (1990) c-fos protooncogene expression and neuronal plasticity. *Acta Neurobiol Exp (Wars)* 50:173–9. Available at: http://www.ncbi.nlm.nih.gov/pubmed/2130639 [Accessed May 26, 2014].

12. Young ST, Porrino LJ, Iadarola MJ (1991) Cocaine induces striatal c-fos-immunoreactive proteins via dopaminergic D1 receptors. *Proc Natl Acad Sci U S A* 88:1291–5. Available at: http://www.pubmedcentral.nih.gov/articlerender.fcgi?artid=51003&tool=pmcentrez&rendertype=abstract [Accessed June 29, 2014].

13. Guo YP et al. (2007) Corticothalamic synchronization leads to c-fos expression in the auditory thalamus. *Proc Natl Acad Sci U S A* 104:11802–7. Available at: http://www.pubmedcentral.nih.gov/articlerender.fcgi?artid=1913871&tool=pmcentrez&rendertype=abstract [Accessed June 29, 2014].

14. Saint Marie RL, Luo L, Ryan AF (1999) Effects of stimulus frequency and intensity on c-fos mRNA expression in the adult rat auditory brainstem. *J Comp Neurol* 404:258–70. Available at: http://www.ncbi.nlm.nih.gov/pubmed/9934998 [Accessed June 29, 2014].

15. Zhang JS, Kaltenbach JA, Wang J, Kim SA (2003) Fos-like immunoreactivity in auditory and nonauditory brain structures of hamsters previously exposed to intense sound. *Exp brain Res* 153:655–60. Available at: http://www.ncbi.nlm.nih.gov/pubmed/12955379 [Accessed June 29, 2014].

16. Cho S et al. (2001) Early c-Fos induction after cerebral ischemia: a possible neuroprotective role. *J Cereb Blood Flow Metab* 21:550–6. Available at: http://www.ncbi.nlm.nih.gov/pubmed/11333365 [Accessed June 29, 2014].

17. Byun K et al. (2013) Changes of calcium binding proteins, c-Fos and COX in hippocampal formation and cerebellum of Niemann-Pick, type C mouse. *J Chem Neuroanat* 52:1–8. Available at: http://www.ncbi.nlm.nih.gov/pubmed/23660496 [Accessed June 29, 2014].

18. Sun H, Charles CH, Lau LF, Tonks NK (1993) MKP-1 (3CH134), an immediate early gene product, is a dual specificity phosphatase that dephosphorylates MAP kinase in vivo. *Cell* 75:487–93. Available at: http://www.ncbi.nlm.nih.gov/pubmed/8221888 [Accessed June 8, 2014].

19. Charles CH, Sun H, Lau LF, Tonks NK (1993) The growth factor-inducible immediate-early gene 3CH134 encodes a protein-tyrosine-phosphatase. *Proc Natl Acad Sci U S A* 90:5292–6. Available at: http://www.pubmedcentral.nih.gov/articlerender.fcgi?artid=46702&tool=pmcentrez&rendertype=abstract [Accessed June 30, 2014].

20. Keyse SM, Emslie EA (1992) Oxidative stress and heat shock induce a human gene encoding a protein-tyrosine phosphatase. *Nature* 359:644–7. Available at: http://www.ncbi.nlm.nih.gov/pubmed/1406996 [Accessed June 30, 2014].

21. Shipp LE et al. (2010) Transcriptional regulation of human dual specificity protein phosphatase 1 (DUSP1) gene by glucocorticoids. *PLoS One* 5:e13754. Available at: http://www.pubmedcentral.nih.gov/articlerender.fcgi?artid=2966426&tool=pmcentrez&rendertype=abstract [Accessed June 30, 2014].

22. King EM, Holden NS, Gong W, Rider CF, Newton R (2009) Inhibition of NF-kappaB-dependent transcription by MKP-1: transcriptional repression by glucocorticoids occurring via p38 MAPK. *J Biol Chem* 284:26803–15. Available at: http://www.pubmedcentral.nih.gov/articlerender.fcgi?artid=2785369&tool=pmcentrez&rendertype=abstract [Accessed June 11, 2014].

23. Kwak SP, Hakes DJ, Martell KJ, Dixon JE (1994) Isolation and characterization of a human dual specificity protein-tyrosine phosphatase gene. *J Biol Chem* 269:3596–604. Available at: http://www.ncbi.nlm.nih.gov/pubmed/8106404 [Accessed June 30, 2014].

24. Vernes SC et al. (2007) High-throughput analysis of promoter occupancy reveals direct neural targets of FOXP2, a gene mutated in speech and language disorders. *Am J Hum Genet* 81:1232–50. Available at: http://www.pubmedcentral.nih.gov/articlerender.fcgi?artid=2276341&tool=pmcentrez&rendertype=abstract [Accessed June 3, 2014].

25. Spiteri E et al. (2007) Identification of the transcriptional targets of FOXP2, a gene linked to speech and language, in developing human brain. *Am J Hum Genet* 81:1144–57. Available at: http://www.pubmedcentral.nih.gov/articlerender.fcgi?artid=2276350&tool=pmcentrez&rendertype=abstract [Accessed June 27, 2014].

26. Del Rosso M et al. (1993) Urokinase-urokinase receptor interaction: non-mitogenic signal transduction in human epidermal cells. *Biochem Biophys Res Commun* 190:347–52. Available at: http://www.ncbi.nlm.nih.gov/pubmed/8381273 [Accessed July 4, 2014].

27. Anichini E et al. (1997) Interaction of urokinase-type plasminogen activator with its receptor rapidly induces activation of glucose transporters. *Biochemistry* 36:3076–83. Available at: http://www.ncbi.nlm.nih.gov/pubmed/9115983 [Accessed July 4, 2014].

28. Goretzki L, Mueller BM (1997) Receptor-mediated endocytosis of urokinase-type plasminogen activator is regulated by cAMP-dependent protein kinase. *J Cell Sci* 110 (Pt 1:1395–402. Available at: http://www.ncbi.nlm.nih.gov/pubmed/9217325 [Accessed July 4, 2014].

29. Cao D et al. (1995) Human urokinase-type plasminogen activator primes neutrophils for superoxide anion release. Possible roles of complement receptor type 3 and calcium. *J Immunol* 154:1817–29. Available at: http://www.ncbi.nlm.nih.gov/pubmed/7836767 [Accessed July 4, 2014].

30. Vilhardt F, Nielsen M, Sandvig K, van Deurs B (1999) Urokinase-type plasminogen activator receptor is internalized by different mechanisms in polarized and nonpolarized Madin-Darby canine kidney epithelial cells. *Mol Biol Cell* 10:179–95. Available at: http://www.pubmedcentral.nih.gov/articlerender.fcgi?artid=25162&tool=pmcentrez&rendertype=abstract [Accessed July 4, 2014].

31. Resnati M et al. (1996) Proteolytic cleavage of the urokinase receptor substitutes for the agonist-induced chemotactic effect. *EMBO J* 15:1572–82. Available at: http://www.pubmedcentral.nih.gov/articlerender.fcgi?artid=450067&tool=pmcentrez&rendertype=abstract [Accessed July 4, 2014].

32. Brodie C et al. (1999) Protein kinase C-epsilon plays a role in neurite outgrowth in response to epidermal growth factor and nerve growth factor in PC12 cells. *Cell Growth Differ* 10:183–91. Available at: http://www.ncbi.nlm.nih.gov/pubmed/10099832 [Accessed July 4, 2014].

33. Wada K et al. (2006) A molecular neuroethological approach for identifying and characterizing a cascade of behaviorally regulated genes. *Proc Natl Acad Sci U S A* 103:15212–7. Available at: http://www.pnas.org/content/103/41/15212.full [Accessed June 12, 2014].

34. Barresi V et al. (2010) Decreased expression of GRAF1/OPHN-1-L in the X-linked alpha thalassemia mental retardation syndrome. *BMC Med Genomics* 3:28. Available at: http://www.pubmedcentral.nih.gov/articlerender.fcgi?artid=2915949&tool=pmcentrez&rendertype=abstract [Accessed July 4, 2014].

35. West AE, Greenberg ME (2011) Neuronal activity-regulated gene transcription in synapse development and cognitive function. *Cold Spring Harb Perspect Biol* 3. Available at: http://www.pubmedcentral.nih.gov/articlerender.fcgi?artid=3098681&tool=pmcentrez&rendertype=abstract [Accessed June 7, 2014].

36. Santoro SW, Dulac C (2012) The activity-dependent histone variant H2BE modulates the life span of olfactory neurons. *Elife* 1:e00070. Available at: http://elifesciences.org/content/1/e00070.abstract [Accessed May 31, 2014].

37. Mello C V, Clayton DF (1994) Song-induced ZENK gene expression in auditory pathways of songbird brain and its relation to the song control system. *J Neurosci* 14:6652–66. Available at: http://www.ncbi.nlm.nih.gov/pubmed/7965067 [Accessed July 4, 2014].

38. Mello C V, Ribeiro S (1998) ZENK protein regulation by song in the brain of songbirds. *J Comp Neurol* 393:426–38. Available at: http://www.ncbi.nlm.nih.gov/pubmed/9550149 [Accessed July 4, 2014].

39. Thode C, Bock J, Braun K, Darlison MG (2005) The chicken immediate-early gene ZENK is expressed in the medio-rostral neostriatum/hyperstriatum ventrale, a brain region involved in acoustic imprinting, and is up-regulated after exposure to an auditory stimulus. *Neuroscience* 130:611–7. Available at: http://www.sciencedirect.com/science/article/pii/S0306452204009558 [Accessed July 4, 2014].

40. Jarvis ED, Nottebohm F (1997) Motor-driven gene expression. *Proc Natl Acad Sci U S A* 94:4097–102. Available at: http://www.pubmedcentral.nih.gov/articlerender.fcgi?artid=20574&tool=pmcentrez&rendertype=abstract [Accessed July 4, 2014].

41. Nuutinen S, Panula P (2011) Histamine in neurotransmission and brain diseases. *Adv Exp Med Biol* 709:95–107. Available at: http://www.ncbi.nlm.nih.gov/pubmed/21713693 [Accessed June 12, 2014].

42. Ercan-Sencicek AG et al. (2010) L-histidine decarboxylase and Tourette’s syndrome. *N Engl J Med* 362:1901–8. Available at: http://www.pubmedcentral.nih.gov/articlerender.fcgi?artid=2894694&tool=pmcentrez&rendertype=abstract [Accessed June 28, 2014].

43. Passani MB, Lin J-S, Hancock A, Crochet S, Blandina P (2004) The histamine H3 receptor as a novel therapeutic target for cognitive and sleep disorders. *Trends Pharmacol Sci* 25:618–25. Available at: http://www.ncbi.nlm.nih.gov/pubmed/15530639 [Accessed June 6, 2014].

44. Wendt KD et al. (2005) Behavioral assessment in mouse models of neuronal ceroid lipofuscinosis using a light-cued T-maze. *Behav Brain Res* 161:175–82. Available at: http://www.ncbi.nlm.nih.gov/pubmed/15885820 [Accessed June 28, 2014].

45. Bolivar VJ, Scott Ganus J, Messer A (2002) The development of behavioral abnormalities in the motor neuron degeneration (mnd) mouse. *Brain Res* 937:74–82. Available at: http://www.ncbi.nlm.nih.gov/pubmed/12020865 [Accessed June 28, 2014].

46. Battaglioli G, Martin DL, Plummer J, Messer A (1993) Synaptosomal glutamate uptake declines progressively in the spinal cord of a mutant mouse with motor neuron disease. *J Neurochem* 60:1567–9. Available at: http://www.ncbi.nlm.nih.gov/pubmed/8095977 [Accessed June 28, 2014].

47. Bigini P et al. (2012) Increased [^{3}H]D-aspartate release and changes in glutamate receptor expression in the hippocampus of the mnd mouse. *J Neurosci Res* 90:1148–58. Available at: http://www.ncbi.nlm.nih.gov/pubmed/22302580 [Accessed June 28, 2014].

48. Kolikova J, Afzalov R, Surin A, Lehesjoki A-E, Khiroug L (2011) Deficient mitochondrial Ca(2+) buffering in the Cln8(mnd) mouse model of neuronal ceroid lipofuscinosis. *Cell Calcium* 50:491–501. Available at: http://www.ncbi.nlm.nih.gov/pubmed/21917311 [Accessed June 28, 2014].

49. Vantaggiato C et al. (2009) A novel CLN8 mutation in late-infantile-onset neuronal ceroid lipofuscinosis (LINCL) reveals aspects of CLN8 neurobiological function. *Hum Mutat* 30:1104–16. Available at: http://www.ncbi.nlm.nih.gov/pubmed/19431184 [Accessed June 28, 2014].

50. Shewan D et al. (1996) mCD24, a glycoprotein transiently expressed by neurons, is an inhibitor of neurite outgrowth. *J Neurosci* 16:2624–34. Available at: http://www.ncbi.nlm.nih.gov/pubmed/8786438 [Accessed July 4, 2014].

51. Calaora V, Chazal G, Nielsen PJ, Rougon G, Moreau H (1996) mCD24 expression in the developing mouse brain and in zones of secondary neurogenesis in the adult. *Neuroscience* 73:581–94. Available at: http://www.ncbi.nlm.nih.gov/pubmed/8783272 [Accessed July 4, 2014].

52. Belvindrah R, Rougon G, Chazal G (2002) Increased neurogenesis in adult mCD24-deficient mice. *J Neurosci* 22:3594–607. Available at: http://www.ncbi.nlm.nih.gov/pubmed/11978835 [Accessed July 4, 2014].

53. Giffard RG et al. (2000) The electrogenic sodium bicarbonate cotransporter: developmental expression in rat brain and possible role in acid vulnerability. *J Neurosci* 20:1001–8. Available at: http://www.ncbi.nlm.nih.gov/pubmed/10648705 [Accessed July 4, 2014].

54. Strømgaard K, Mellor I (2004) AMPA receptor ligands: synthetic and pharmacological studies of polyamines and polyamine toxins. *Med Res Rev* 24:589–620. Available at: http://www.ncbi.nlm.nih.gov/pubmed/15224382 [Accessed June 29, 2014].

55. Mony L, Kew JNC, Gunthorpe MJ, Paoletti P (2009) Allosteric modulators of NR2B-containing NMDA receptors: molecular mechanisms and therapeutic potential. *Br J Pharmacol* 157:1301–17. Available at: http://www.pubmedcentral.nih.gov/articlerender.fcgi?artid=2765303&tool=pmcentrez&rendertype=abstract [Accessed May 27, 2014].

56. Zhao Z, Nair SM, Chou DK, Tobet SA, Jungalwala FB (2000) Expression and role of sulfoglucuronyl (HNK-1) carbohydrate and its binding protein SBP-1 in developing rat cerebral cortex. *J Neurosci Res* 62:186–205. Available at: http://www.ncbi.nlm.nih.gov/pubmed/11020212 [Accessed July 4, 2014].

57. Chana G et al. (2013) Biomarker investigations related to pathophysiological pathways in schizophrenia and psychosis. *Front Cell Neurosci* 7:95. Available at: http://www.pubmedcentral.nih.gov/articlerender.fcgi?artid=3693064&tool=pmcentrez&rendertype=abstract [Accessed June 16, 2014].

58. Rouault TA (2013) Iron metabolism in the CNS: implications for neurodegenerative diseases. *Nat Rev Neurosci* 14:551–64. Available at: http://www.ncbi.nlm.nih.gov/pubmed/23820773 [Accessed May 27, 2014].

59. Kadish I et al. (2009) Hippocampal and cognitive aging across the lifespan: a bioenergetic shift precedes and increased cholesterol trafficking parallels memory impairment. *J Neurosci* 29:1805–16. Available at: http://www.pubmedcentral.nih.gov/articlerender.fcgi?artid=2661568&tool=pmcentrez&rendertype=abstract [Accessed June 18, 2014].

60. Chan K-H et al. (2012) Adiponectin is protective against oxidative stress induced cytotoxicity in amyloid-beta neurotoxicity. *PLoS One* 7:e52354. Available at: http://www.pubmedcentral.nih.gov/articlerender.fcgi?artid=3531475&tool=pmcentrez&rendertype=abstract [Accessed July 4, 2014].

61. Song J, Lee WT, Park KA, Lee JE (2014) Association between Risk Factors for Vascular Dementia and Adiponectin. *Biomed Res Int* 2014:261672. Available at: http://www.pubmedcentral.nih.gov/articlerender.fcgi?artid=4016875&tool=pmcentrez&rendertype=abstract [Accessed June 28, 2014].

62. Zhao T et al. (2012) Dopaminergic neuronal loss and dopamine-dependent locomotor defects in Fbxo7-deficient zebrafish. *PLoS One* 7:e48911. Available at: http://www.pubmedcentral.nih.gov/articlerender.fcgi?artid=3487786&tool=pmcentrez&rendertype=abstract [Accessed July 4, 2014].

63. Kuiken HJ et al. (2012) Identification of F-box only protein 7 as a negative regulator of NF-kappaB signalling. *J Cell Mol Med* 16:2140–9. Available at: http://www.ncbi.nlm.nih.gov/pubmed/22212761 [Accessed June 24, 2014].

64. Zhao T et al. (2013) FBXO7 immunoreactivity in α-synuclein-containing inclusions in Parkinson disease and multiple system atrophy. *J Neuropathol Exp Neurol* 72:482–8. Available at: http://www.ncbi.nlm.nih.gov/pubmed/23656991 [Accessed June 27, 2014].

65. Stopkova P et al. (2003) Polymorphism screening of PIP5K2A: a candidate gene for chromosome 10p-linked psychiatric disorders. *Am J Med Genet B Neuropsychiatr Genet* 123B:50–8. Available at: http://www.ncbi.nlm.nih.gov/pubmed/14582145 [Accessed July 5, 2014].

66. Shyng SL et al. (2000) Modulation of nucleotide sensitivity of ATP-sensitive potassium channels by phosphatidylinositol-4-phosphate 5-kinase. *Proc Natl Acad Sci U S A* 97:937–41. Available at: http://www.pubmedcentral.nih.gov/articlerender.fcgi?artid=15434&tool=pmcentrez&rendertype=abstract [Accessed July 5, 2014].

67. Chatah NE, Abrams CS (2001) G-protein-coupled receptor activation induces the membrane translocation and activation of phosphatidylinositol-4-phosphate 5-kinase I alpha by a Rac- and Rho-dependent pathway. *J Biol Chem* 276:34059–65. Available at: http://www.ncbi.nlm.nih.gov/pubmed/11431481 [Accessed July 5, 2014].

68. Wenk MR et al. (2001) PIP kinase Igamma is the major PI(4,5)P(2) synthesizing enzyme at the synapse. *Neuron* 32:79–88. Available at: http://www.ncbi.nlm.nih.gov/pubmed/11604140 [Accessed July 5, 2014].

69. Clarke JH, Irvine RF (2013) Enzyme activity of the PIP4K2A gene product polymorphism that is implicated in schizophrenia. *Psychopharmacology (Berl)* 230:329–31. Available at: http://www.pubmedcentral.nih.gov/articlerender.fcgi?artid=3825611&tool=pmcentrez&rendertype=abstract [Accessed June 9, 2014].

70. Thiselton DL et al. (2010) Association analysis of the PIP4K2A gene on chromosome 10p12 and schizophrenia in the Irish study of high density schizophrenia families (ISHDSF) and the Irish case-control study of schizophrenia (ICCSS). *Am J Med Genet B Neuropsychiatr Genet* 153B:323–31. Available at: /pmcc/articles/PMC4011176/?report=abstract [Accessed July 5, 2014].

71. Fedorenko O et al. (2009) PIP5K2A-dependent regulation of excitatory amino acid transporter EAAT3. *Psychopharmacology (Berl)* 206:429–35. Available at: http://www.ncbi.nlm.nih.gov/pubmed/19644675 [Accessed July 5, 2014].

72. Fedorenko O et al. (2008) A schizophrenia-linked mutation in PIP5K2A fails to activate neuronal M channels. *Psychopharmacology (Berl)* 199:47–54. Available at: http://www.ncbi.nlm.nih.gov/pubmed/18545987 [Accessed July 5, 2014].

73. Ahn J-H et al. (2007) The B''/PR72 subunit mediates Ca2+-dependent dephosphorylation of DARPP-32 by protein phosphatase 2A. *Proc Natl Acad Sci U S A* 104:9876–81. Available at: http://www.pubmedcentral.nih.gov/articlerender.fcgi?artid=1887582&tool=pmcentrez&rendertype=abstract [Accessed June 19, 2014].

74. Papadia S et al. (2008) Synaptic NMDA receptor activity boosts intrinsic antioxidant defenses. *Nat Neurosci* 11:476–87. Available at: http://www.pubmedcentral.nih.gov/articlerender.fcgi?artid=2556874&tool=pmcentrez&rendertype=abstract [Accessed May 28, 2014].

75. Soriano FX, Papadia S, Bell KFS, Hardingham GE (2009) Role of histone acetylation in the activity-dependent regulation of sulfiredoxin and sestrin 2. *Epigenetics* 4:152–8. Available at: http://www.pubmedcentral.nih.gov/articlerender.fcgi?artid=2830533&tool=pmcentrez&rendertype=abstract [Accessed July 5, 2014].

76. Jung D-J et al. (2002) Novel transcription coactivator complex containing activating signal cointegrator 1. *Mol Cell Biol* 22:5203–11. Available at: http://www.pubmedcentral.nih.gov/articlerender.fcgi?artid=139772&tool=pmcentrez&rendertype=abstract [Accessed July 4, 2014].

77. Smith DJ et al. (1997) Functional screening of 2 Mb of human chromosome 21q22.2 in transgenic mice implicates minibrain in learning defects associated with Down syndrome. *Nat Genet* 16:28–36. Available at: http://www.ncbi.nlm.nih.gov/pubmed/9140392 [Accessed June 14, 2014].

78. Golden JA, Hyman BT (1994) Development of the superior temporal neocortex is anomalous in trisomy 21. *J Neuropathol Exp Neurol* 53:513–20. Available at: http://www.ncbi.nlm.nih.gov/pubmed/8083693 [Accessed July 5, 2014].

79. Raz N et al. (1995) Selective neuroanatomic abnormalities in Down's syndrome and their cognitive correlates: evidence from MRI morphometry. *Neurology* 45:356–66. Available at: http://www.ncbi.nlm.nih.gov/pubmed/7854539 [Accessed July 1, 2014].

80. Lyle R, Gehrig C, Neergaard-Henrichsen C, Deutsch S, Antonarakis SE (2004) Gene expression from the aneuploid chromosome in a trisomy mouse model of down syndrome. *Genome Res* 14:1268–74. Available at: http://www.pubmedcentral.nih.gov/articlerender.fcgi?artid=442141&tool=pmcentrez&rendertype=abstract [Accessed June 14, 2014].

81. Di Benedetto D et al. (2013) 6p22.3 deletion: report of a patient with autism, severe intellectual disability and electroencephalographic anomalies. *Mol Cytogenet* 6:4. Available at: http://www.pubmedcentral.nih.gov/articlerender.fcgi?artid=3564794&tool=pmcentrez&rendertype=abstract [Accessed June 5, 2014].

82. Common genetic variation and antidepressant efficacy in major depressive disorder: a meta-analysis of three genome-wide pharmacogenetic studies. (2013) *Am J Psychiatry* 170:207–17. Available at: http://www.ncbi.nlm.nih.gov/pubmed/23377640 [Accessed July 2, 2014].

83. Ludwig KU (2009) Molecular genetic analyses in developmental dyslexia and related endophenotypes. *Dissertation*. Available at: http://hss.ulb.uni-bonn.de/2010/2372/2372.pdf [Accessed July 4, 2014].

84. Higa LA et al. (2006) CUL4-DDB1 ubiquitin ligase interacts with multiple WD40-repeat proteins and regulates histone methylation. *Nat Cell Biol* 8:1277–83. Available at: http://www.ncbi.nlm.nih.gov/pubmed/17041588 [Accessed July 15, 2014].

85. Chen C-Y et al. (2012) Rescue of the genetically engineered Cul4b mutant mouse as a potential model for human X-linked mental retardation. *Hum Mol Genet* 21:4270–85. Available at: http://www.ncbi.nlm.nih.gov/pubmed/22763239 [Accessed July 15, 2014].
